# Activities of daily living and quality of life in Alzheimer disease

**Published:** 2012-06-18

**Authors:** JA Opara

**Affiliations:** Academy of Physical Education in Katowice, Poland

**Keywords:** Alzheimer’s Disease, ADL, burden, IADL, Quality of Life

## Abstract

Alzheimer’s disease is known for placing a significant burden on caregivers, which includes social, psychological, physical or economic aspects. The disease decreases patients’ capacity for activities of daily living and quality of life. Information about functional status is useful in the interpretation of the quality of life assessment results.

In this paper the most commonly used scales evaluating activities of daily living and quality of life in Alzheimer’s disease, either generic or specific, is presented.

## Introduction

Quality of Life (QoL) is a multi-dimensional construct, which consists of at least three broad domains: physical, mental and social. In the field of medicine, researchers and physicians have often used health-related quality of life concepts, which specifically focus on the impact of an illness and/or treatment on patients’ perception of their status of health and on subjective well-being or satisfaction with life [**1**]. In our first report, the Quality of Life of post-stroke patients and their caregivers was described [**2**]. The Quality of Life of patients with Multiple Sclerosis was described in our second review [**3**].

More than two-thirds of persons suffering from Alzheimer’s disease stay at home, being taken care after by family and friends. Their illness has a significant impact on their families. As the disease advances, symptoms include confusion, irritability and aggression, mood swings, language breakdown, long-term memory loss, and the general withdrawal as senses decline. Gradually, bodily functions are lost [**4**]. The disease diminishes patients` independence in Activities of Daily Living (ADL) and quality of life (QoL). The quality of life of carers also deteriorates. 

Quality of life (QoL) in dementing illness comprises the same areas as in the general population. Domains of QoL in patients with Alzheimer’s disease (AD) include competent cognitive functioning, the ability to perform Activities of Daily Living (ADL) and to engage in meaningful use of time and social behavior, and a favorable balance between positive emotions and absence of negative emotions [**5**].

The spouse or a close relative often takes the role of the main caregiver. Alzheimer's disease is known for placing a great burden on caregivers, which includes social, psychological, physical and economic aspects. Patients and families usually prefer homecare [**6**]. Dementia caregivers are subject to high rates of physical and mental disorders. Their quality of life is usually diminished.

The methods of assessment of activities of daily living, as well as quality of life of Alzheimer’s disease sufferers and their carers will be described in this paper.

### 1. Activities of Daily Living in AD

The assessment of ADL in Alzheimer’s Disease (AD) is necessary for the evaluation of the patient`s needs, which entails the cost and burden on caregivers. For the evaluation of ADL in dementia, the commonly known scales are Barthel Index (BI), Katz Index and the Functional Independence Measure (FIM).

These are three specific scales used to assess basic ADL in dementia: the Blessed ADL scale, the Bristol Scale and the Functional Activities Questionnaire. 

ADL in Dementia Scale (Blessed ADL Scale, used as a shortcut DS) was described by the Blessed, Tomlinson and Roth in 1965. In the modified version, from 1988, by Erkinjuntti et al, it consists of 13 items divided into two parts: the first part consisting of 10 items, the system of points 0, 0.5 and 1, and the second part is to choose four options scored from 0 to 3 [**7**].

The Bristol ADL scale was described by Hughes et al. in 1982. In 1996, the modified version by Bucks et al. consists of 14 items carried out in a four-scale from 0 to 3 points [**8**]. Currently, it contains 20 items. 

In 1982, Pfeffer et al. described the Functional Activities Questionnaire (FAQ). It was formed by selecting the most representative of dementia ten steps Lawton Instrumental ADL Scale. The evaluation system ranges from 0 to 3 points - the total score in the FAQ ranges from 0 points (independent) to 30 points (dependent) [**9**]**[appendix I]**.

In 1999, Gelinas et al created a scale for the Disability Assessment for Dementia (DAD). It assesses in the simplest zero-one (yes or no) system 40 operations, 17 of which are associated with basic ADL and 23 with instrumental ADL [**10**].

### 2. Instrumental Activities of Daily Living in AD

To assess more complex daily activities in dementia the Instrumental (Extended) Activities of Daily Living Scales are used. IADL (or EADL) scales are situated in between ADL scales and questionnaires measuring Quality of Life. A systematic review of the ADL and IADL scales used for diagnosis and treatment of dementia is found in Sjetske Sikkes et al. [**11**]. After a selection, they evaluated a total of 12 scales. Two scales of ADL: ADL, DAD and Bristol received two positive reviews and were classified as moderate quality. The five scales: Activities of Daily Living Prevention Instrument (ADL-PI), Alzheimer's Disease Activities of Daily Living International Scale (ADL-IS), Bayer Activities of Daily Living Scale (B-ADL), the Cleveland Scale for Activities of Daily Living (CSADL) and the Lawton IADL received a positive evaluation. Interpretation: The results indicate that, in order to justify their use, it is necessary to improve them and to have more data on the psychometric properties of ADL scales in dementia.

Questionnaire Activities of Daily Living Prevention Instrument (ADL-PI) consists of 20 items [**12**]. Alzheimer Disease Activities of Daily Living International Scale (ADL-IS) consists of 40 items [**13**]. Bayer Activities of Daily Living Scale (B-ADL) consists of 25 items [**14**]. Cleveland Scale for Activities of Daily Living (CSADL) consists of 47 items [**15**]. Lawton & Brody Instrumental ADL consists of eight items [**16**].

### 3. Quality of Life in AD

Quality of Life (QoL) is a multi-dimensional construct, which consists of at least three broad domains: physical, mental and social. In the field of medicine, researchers and physicians have often used health-related quality of life concepts, which specifically focus on the impact of an illness and/or treatment on patients’ perception of their status of health and on subjective well-being or satisfaction with life [**1**]. The Quality of Life of post-stroke patients and their caregivers was described in our first report [**2**]. The Quality of Life of patients with Multiple Sclerosis was described in the next review [**3**].

AD can cause a variety of symptoms, including changes in mood, cognitive impairment, problems with balance, incontinence, insomnia, confusion, irritability, aggression, mood swings, etc. AD will cause impaired mobility and disability in more severe cases. Alzheimer’s disease may develop quickly or slowly over time. Permanent problems often persist, especially as the disease advances. AD does not currently have a cure, though several treatments are available, that may slow the course of disease.

QoL scales for patients suffering from AD could be divided into universal (general - generic) and specific for the disease (disease - oriented). 

#### 3.A. Generic questionnaires

Among the generic questionnaires used in other disease entities, the assessment of QoL in patients with AD, which are mostly used are Medical Outcome Study 36-Item Short Form Health Survey - SF - 36, EuroQol EQ-5D, Sickness Impact Profile (SIP), Life Satisfaction Questionnaire - LSQ, WHOQOL BREF and Quality of Well-Being Scale – QWBS. 

The above-mentioned questionnaires have been tested in many countries. There are numerous and detailed data in the literature on their validity and reliability. The scale of the SF-36 allows the assessment of the eight areas of QoL during the four weeks preceding the survey, completion taking about 9 mins. It is particularly useful in predicting the course of the disease [**17**]. The disadvantages include the effect of the lower and upper limit and a relatively low sensitivity to change.

The scale EQ-5D allows the assessment of the five areas of health and self-esteem at the time of the study. Completion time is of 3 mins. Because of the three levels evaluation system, EQ-5D is poorly sensitive to changes in QoL, especially in patients with a score of 5 and over by EDSS. It is primarily intended for the management of healthcare and for healthcare decision-makers [**18**].

SIP questionnaire allows the assessment of 12 areas of functioning at the time of study and (in contrast to SF-36 and EQ-5D) it is sensitive to the patient`s change. The disadvantages of SIP include its length (136 items) which makes the completion time be up to 30 minutes [**19**]. 

Life Satisfaction Questionnaire – LSQ - originally developed by Carlsson and Hamrin for the assessment of the quality of life for women after mastectomy, started being used in various other diseases. The modified version is made up of 67 questions covering nine areas of life (overall living situation, daily activities - ADL, social life, family relationships, health evaluation, finance, employment, drugs, problem solving) and overall satisfaction with life. The patients may express their views in a 7-point scale from 1 (extreme dissatisfaction) to 7 (complete satisfaction). Point discrimination "satisfaction" and "discontent" in the questionnaire LSQ is between 3 and 5 - a score of 4 or more is regarded as an indicator of satisfaction [**20**]. 

The Scale of the Welfare Quality (Quality of Well-Being Scale - QWBS) allows the assessment of mobility, physical activity, social activity and 27 symptoms. The combination of the above categories can identify 43 patient's functional levels. It is recommended, that a trained person complete the questionnaire during an interview. Completion time is between 10 - 15 min [**21**]. 

In 1961, Neugarten et al. described the Life Satisfaction Index-A (LSI-A). It consists of 18 questions, to which the respondent can answer "yes" or "no". In 1969, a modified version by Wood and colleagues referred to the sense of aging - the Life Satisfaction Index Z - Life Satisfaction Index-Z (LSI-Z) consists of 13 questions [**22**]. 

Summary of Individual Quality of Life Assessment - Schedule of the Evaluation of Individual Quality of Life (SEIQoL) was described by Browne et al., in 1994 [**23**]. In the directly balanced version - Schedule of the Evaluation of Individual Quality of Life Direct Weighting (SEIQoL-DW) studies were performed on patients over 65 years of age. Test SEIQoL-DW was too difficult and time-consuming for most patients. However, it can be a valuable addition to knowledge for staff caring for elderly people [**24**]. 

LEIPAD Scale took its name from two places: Leiden and Padua. It consists of 49 questions covering seven sub-scales: physical functioning, personal care, depression and anxiety, cognitive function, social function, sexual function, overall satisfaction with life [**25**]. 

#### 3.B. Questionnaires specific for AD

Dementia caregivers are subject to high rates of physical and mental disorders. Factors associated with greater psychosocial problems of the primary caregivers include having an affected person at home, the carer being a spouse, demanding behaviors of the cared person such as depression, behavioral disturbances, hallucinations, sleep problems or walking disruption and social isolation. Regarding economic problems, family caregivers often give up time from work to spend 47 hours per week on average with the person with AD, while the costs of caring for them are high [**26,27**]. Inouye and colleagues have systematically reviewed the literature regarding the quality of life in Alzheimer's disease (AD). They found 10 AD-specific questionnaires: 

1) QUALIDEM, 2) Psychological Well-Being in Cognitively Impaired Persons (PWB-CIP), 3) DEMQOL, 4) Quality of Life Assessment Scale on Alzheimer’s Disease (QoL-AD), 5) The Cornell-Brown Scale for Quality of Life in Dementia (CBS), 6) Dementia Care Mapping (DCM), 7) Quality of Life Assessment Schedule in Dementia (QOLAS), 8) Quality of Life in Late-Stage Dementia Scale (QUALID), 9) Dementia Quality of Life Instrument (DQOL) and 10) Activity and Affect Indicators of Quality of Life. Five of them: QUALIDEM, PWB-CIP, DEMQOL, DCM, QUALID Activity and Affect Indicators of Quality of Life, produced based on the carers’ reports, are suitable for evaluation of severe cases of AD, while DEMQOL, QDV-DA, CBS, and QOLAS DQOL as completed by patients, can be used to assess quality of life in the early stages of the disease. It follows that the most comprehensive scale is the DEMQOL [**28**].

In 1999, Rabins and colleagues published a questionnaire related quality of life of Alzheimer's Disease - Alzheimer's Disease-Related Quality of Life (ADRQL). The modified version is made up of 40 items covering five sub-scales: social interaction, self-awareness, feelings and moods, pleasure and response to environment [**29**].

In 2005, Smith et al. have published two questionnaires DEMQOL, designed to assess the quality of life associated with dementia. DEMQOL questionnaire consists of 29 questions and DEMQOL-Proxy from 32 matches. The self-assessment questionnaire, DEMQOL, is particularly useful for the assessment of the quality of life associated with the disease (HRQOL), of people with mild to moderate dementia, but it is not appropriate for patients with advanced dementia (Mini Mental State Examination - MMSE < 10). Questionnaire DEMQOL-Proxy is useful to evaluate the quality of life of the relatives or carers (proxies) of patients with both mild and moderate dementia, as well as severe dementia (MMSE ≥ 10). DEMQOL questionnaires consist of three main parts: questions about the previous week, memory and activities of daily living (ADL), while the last two questions relate to general health and overall quality of life [**30**].

#### 3.C. Questionnaires evaluating burden and QoL of carers

There are many different definitions of the burden of taking care of the chronically ill (burden, strain, stress) in the literature. Pearlin and colleagues refer to them as problems of physical, mental, emotional, social and financial functioning [**31**]. A close family member, often a spouse or child, usually a daughter, who lives with the parent, usually provides assistance. Carers provide basic personal hygiene, assistance with daily activities, provide emotional support, and arrange for medical services and social assistance. Caring for a patient may have an impact on the objective and subjective aspects of the carer, such as physical and emotional health, morale, job, finances, social activity, relationships and sex life. In studies assessing the psychological consequences of care, a higher level of anxiety and depression in caregivers than in the general population has been found. 

**3.C.1.** The most commonly used generic questionnaires about the quality of life of caregivers are: Burden Interview (BI index), Caregiver Burden Scale (CBS), Caregiver Reaction Assessment (CRA), Caregiver Strain Index (CS), Life Situation Questionnaire (LSQ), the Sense of Competence Questionnaire (SCQ), 26-item WHOQOL-Bref, SF-36 Health Survey and the Zarit Caregiver Burden Interview (ZCBI) [**16,32,33**]. The latter consists of 22 items assessing the impact of disability on the patient's physical health and emotional state, and social and financial repercussions. The range of scoring is from never (0 points) to almost always (score 4). The final evaluation after summing the individual is from 0 to 88. The higher the score, the higher the perceived burden on the carer - CB (caregivers burden) [**34**]. 

In 1989, Novak and Guest described the Burden Inventory Supervisor - Caregiver Burden Inventory (CBI). This simple and easy to understand questionnaire consists of 24 questions included in the five sub-scales. These are the following: time spent on care, psychological stress, physical stress, social burden (feeling bad relationships in the family and at work) and emotional burden (negative feelings towards the patient). Possible answers: 0 = not at all, 1 = little, 2 = medium, 3 - 4 = a lot of [**35**]. 

**3.C.2.** Schulz et al. conducted a systematic review of reports on quality of life for carers of people with dementia in 2002 (Dementia caregiver intervention research: in search of clinical significance. Gerontologist 2002;42,5:589-602). They discussed the most frequently used questionnaires: Caregiver (Caregiving) Appraisal (Lawton, Kleban, Moss, Rovine, & Glickman, 1989), Perceived Caregiver Burden Scale (Given, Stommel, Collins, & King, 1990), Caregiver Task Checklist (Poulshock & Deimling, 1984), Consequences of Caregiving Memory and Behavior Problem Checklist (Zarit, Reever, & Bach-Peterson, 1980), Caregiver Burden Scale (Carey, Oberst, McCubbin, & Hughes, 1991; Oberst, Thomas, Gass, & Ward, 1989) , Caregiver Distress Scale Screen for Caregiver Burden (Vitaliano, Russo, Young, Becker, & Maiuro, 1991), the Zarit Burden Interview (Zarit et al., 1980), Objective and subjective Burden (Montgomery, Gonyea, & Hooyman, 1985) and Revised Burden Interview (Zarit, Orr, & Zarit, 1987) [**36**]. To assess the quality of life of caregivers the following are also used: Life Satisfaction Index-A (LSI-A), Life Satisfaction Index-Z (LSI-Z), Schedule of the Evaluation of Individual Quality of Life Direct Weighting (SEIQoL -DW) and LEIPAD.

There are a few questionnaires specific for the measurement of QoL of carers in Alzheimer disease. Estimates of Care Questionnaire - Caregiving Appraisal was created in 1989 by Lawton et al. The current version consists of 41 questions on health impact on the feelings of the caregiver and his work [**37**]. 

The scale of perceived burden Guardian - Perceived Caregiver Burden Scale (PCBs) - a 31-point scale developed by Stommel et al. in 1990. Each element is assessed by a 4-point scale from 1 = strongly disagree to 4 = fully agree [**38**]. 

Carer Task List - Caregiver (Caregiving) counts Task Checklist the number of hours per week allocated to the execution of 31 tasks related to care [**39**]. 

Questionnaire for Sieve worries Guardian - Caregiver Distress Scale Screen for Caregiver Burden (SCB) - is used to identify poor experiences in the carer of a person suffering from Alzheimer's disease (AD). It consists of 25 items for the evaluation of subjective and objective burden on four elements. They are the following: grief (distress), exposure to stress, ranliwość (vulnerability) and support (resources) [**40**]. 

In 2001, Bédard et al. described a modified, shortened, screening - version of Zarit Burden Interview (ZBI). The abbreviated version consists of 12 elements, and the screening version of 4 elements. Statistical analysis showed full usability of both versions, which is comparable to the full, 22-element version of ZBI [**40**]. 

**Appendix I.** Functional Activities Questionnaire – FAQ 

1. Writing Cheques 

2. Handling Tax and Other Papers 

3. Shop Alone 

4. Heat Water for Coffee, Tea 

5. Preparing Meals 

6. Games of Skill 

7. Current Events 

8. Paying Attention 

9. Remembering Appointments 

10. Travelling Alone

**Acknowledgment: **many thanks to Prof. Michael P. Barnes from Newcastle upon Tyne for revising the text.
